# Constitutive Atg5 overexpression in mouse bone marrow endothelial progenitor cells improves experimental acute kidney injury

**DOI:** 10.1186/s12882-020-02149-1

**Published:** 2020-11-23

**Authors:** Daniel Patschan, Katrin Schwarze, Björn Tampe, Jan Ulrich Becker, Samy Hakroush, Oliver Ritter, Susann Patschan, Gerhard Anton Müller

**Affiliations:** 1 Zentrum für Innere Medizin 1 – Kardiologie, Angiologie, Nephrologie, Klinikum Brandenburg, Medizinische Hochschule Brandenburg, Klinikum Brandenburg, Hochstraße 29, 14770 Brandenburg, Germany; 2 Klinik für Nephrologie und Rheumatologie, Universitätsmedizin Göttingen, Göttingen, Germany; 3 Institut für Allgemeine Pathologie und Pathologische Anatomie, Universitätsklinikum Köln, Köln, Germany; 4 Institut für Pathologie, Universitätsmedizin Göttingen, Göttingen, Germany

**Keywords:** Autophagy, Atg5, EPCs, AKI

## Abstract

**Background:**

Endothelial Progenitor Cells have been shown as effective tool in experimental AKI. Several pharmacological strategies for improving EPC-mediated AKI protection were identified in recent years. Aim of the current study was to analyze consequences of constitutive Atg5 activation in murine EPCs, utilized for AKI therapy.

**Methods:**

Ischemic AKI was induced in male C57/Bl6N mice. Cultured murine EPCs were systemically injected post-ischemia, either natively or after Atg5 transfection (Adenovirus-based approach). Mice were analyzed 48 h and 6 weeks later.

**Results:**

Both, native and transfected EPCs (EPCs^Atg5^) improved persisting kidney dysfunction at week 6, such effects were more pronounced after injecting EPCs^Atg5^. While matrix deposition and mesenchymal transdifferentiation of endothelial cells remained unaffected by cell therapy, EPCs, particularly EPCs^Atg5^ completely prevented the post-ischemic loss of peritubular capillaries. The cells finally augmented the augophagocytic flux in endothelial cells.

**Conclusions:**

Constitutive Atg5 activation augments AKI-protective effects of murine EPCs. The exact clinical consequences need to be determined.

## Background

Syngeneic Proaniogenic Cells (PACs) have been proven as an effective tool in experimental AKI [1–5]. The cells, which in previous years were defined as early Endothelial Progenitor Cells (eEPCs or simply EPCs as oposed to Endothelial Colony Forming Cells – ECFCs; for further references see [6, 7]), have repeatedly been applied in murine AKI, therapeutic effects were robust [1–5]. Own studies performed in the past focused on pharmacological strategies suitable for improving PAC (EPC) mediated AKI protection. Numerous substances with agonistic potency were identified [3, 4, 8–10]. Two of our studies addressed the hypothesis that autophagy activation in PACs can increase renoprotective effects of the cells. The term autophagy (AP) describes a process of intracellular protein degradation, it happens under physiological and non-physiological conditions. Certain stimuli, such as substrate deprivation, can augment AP and thus potentially prolong the lifespan of cells [11]. The so-called autophagocytic cascade involves the proteolytic activation of numerous proteins, the Atgs (Autophagy-related proteins) [12]. Meanwhile more than 30 members of the Atg family have been identified. We do not intend to review the highly complex cascade of AP initiation now. However, in the past we attempted to activate AP in syngeneic PACs by pharmacological measures. In one study, the mediators SuberoylAnilide Hydroxamic Acid (SAHA) and Temsirolimus were employed [13], the second study involved the use of MG-132 (proteasome inhibitor) and of zVAD (pan-caspase inhibitor) [14], respectively. Both studies, of which the second one was performed in diabetic mice, failed to show sustained beneficial effects of pharmacological preconditioning. The reasons for these findings are speculative in nature. One potential explanation is a lack of specificity, the drugs which were applied may have activated pathways not exclusively involved in AP. We therefore aimed to stimulate AP in a more specific manner. In the current study we selectively overexpressed the protein Atg5 in murine PACs, the latter were subsequently injected in post-ischemic mice. Regarding the highly complex dynamics of Atg interactions, one may ask why we decided to exclusively target the protein Atg5. The in vitro deletion of Atg5 has been reported to diminish the endothelial release of the von Willebrand factor [15]. In addition Atg5 inhibition has been shown to reduce the competence of rat EPCs [16]. Finally, a lack of the protein has been documented to aggravate vascular pathology in experimental diabetic nephropathy [17]. Together, these findings clearly suggest a pro-endothelial role of Atg5.

Since we employed a commercially available cell line in our study, the cells will termed EPCs throughout the article.

## Methods

### Atg5 transfection of murine EPCs

Murine EPCs (C57Bl/6 mouse bone marrow progenitor endothelial Cells; Cellbiologics®, C57–6031) were cultured according to the manufacturer’s protocol. In general, cell transduction was performed using the ViraDuctin™ Adenovirus Transduction Reagent (Cell Biolabs®). Thus, the procedure was performed according to the manufacturer’s instructions. One day before transduction, cells were trypsinized and counted. Subsequently, 2 × 10^5^ cells were suspended in 2–3 mL of medium (6-well plate) and incubated at 37 °C overnight. At the day of the transduction, we followed the protocol as mentioned. After finishing the procedure, 1 × 10^6^ cells were concentrated in 100 μL of medium (M1168, Cell Biologics®), respectively, and used for systemic administration in post-ischemic animals. One transduction series allowed to harvest cells for a total number of 10 mice. At the end of every transduction series, a representative number of cells was investigated for red fluorescence in order to confirm Atg5 expression.

### Animal model and surgery

As usual in all of our previous animal-based studies, all protocols were performed according to the guidelines of the German Institute of Health Guide for the Care and Use of Laboratory Animals and approved by the Institutional Animal Care and Use Committee of the University of Göttingen. As in previous mouse studies, we employed male C57/Bl6N mice (8–12 weeks old). Animals were bred in the local animal facility of the Göttingen University Hospital. Mice were separately caged with a 12:12-h light-dark cycle and had free access to water and chow throughout the study. For anesthesia, the following components were administered intraperitoneally: 300 μL 6 mg/100 g ketamine hydrochloride plus 0.77 mg/100 g of xylazine hydrochloride. Animals were subsequently placed on a heated surgical pad and kept there during the whole procedure. Rectal temperature was maintained at 37 °C throughout. The abdomen was opened (1.5-cm midlaparotomy) and both renal pedicles were clamped using microserrefines (Fine Science Tools, Forster City, USA) for 40 min, respectively. In recent studies, cell treatment effects became detectable in those animals which underwent renal pedicle clamping for 40 min, respectively. After 35 min, renal function was not impaired in a consistent manner and 45 min were often associated with an inadequately high mortality. Thus, we decided to exclusively apply 40 min. After releasing the clamps, a constant volume (100 μl) of PAC-containing Endothelial Growth Basal Medium-2 (EBM-2 - Clonetics, Lonza, Walkersville, MD, USA) was injected into the systemic circulation via the tail vein. The respective cell number per injection was 1 × 10^6^. We decided to inject cells at the beginning of reperfusion since at this moment the processes that mediate the so-called reperfusion injury are initiated. Once, the latter have been established, further vascular obstruction evolves and cell homing into the perivascular micorenvironment declines even further. The abdominal incision was closed with a 4–0 suture and surgical staples. In each experimental group 10 animals were analyzed. Animals were sacrificed at 48 h and 6 weeks respectively. Euthanization was performed by injecting the threefold dose of anaethesia (900 μL of 6 mg/100 g ketamine hydrochloride plus 0.77 mg/100 g of xylazine hydrochloride), followed by dissecting the diaphragm. All groups (control and experimental) consisted of *n* = 10 animals, respectively.

### Serum cystatin C

Serum Cystatin C was quantified using a commercially available kit (BioVendor, RD291009200R) according to the manufacturer’s instructions.

### Serum creatinine and blood urea nitrogen (BUN)

Serum creatinine concentration was measured using a commercially available kit (Creatinin, Jaffé, Labor und Technik, Eberhard Lehmann, LT-CR0121, Berlin, Germa-ny) according to the manufacturer’s protocol. BUN analyzes were performed in the laboratory core facility of the university hospital of Göttingen.

### Proteinuria

For measuring proteinuria, animals were held in metabolic cages for 12 h, urine was collected over the whole period. Twenty mL of urine were mixed with 980 μL of Bradford reagent and incubated at room temperature for 10 min. Subsequently, probes were measured at a wave length of 595 nm. Proteinuria was calculated by mg per mL urine/day and finally given as mg/day.

### Periodic acid Schiff (PAS) staining

Conventional pathological investigation was performed in PAS-stained tissue sections. The staining procedure was performed as published previously [4].

### Masson trichrome staining

Masson trichrome staining was performed as in several studies published previously [13, 14, 18]. Briefly, 5 cortical view fields were analyzed per individual kidney, the magnification was × 40, respectively. Glomeruli were excluded. Since interstitial matrix deposition appeared in the color blue, we defined a certain range of blue for color selection. The number of pixels within the pre-defined blueish color range was related to the number of pixels of the whole area investigated. Thus, the results were acquired in percent. The final results were given as relative values, without any unit. Image J software was used for all analyzes.

### Immunofluorescence analyzes

The methods used for immunofluorescence staining have been published previously [19]. However, the essential steps shall be outlined briefly. In general, EndoMT was evaluated in sections stained for CD31 and alpha-Smooth Muscle Actin (aSMA), the endothelial expression of p62 was quantified after co-staining of CD31 and p62. The peritubular capillary density (PTCD) was analyzed in sections stained for CD31 alone. Formalin fixated, paraffin-embedded tissue sections were deparaffinized, followed by incubation in 3% H_2_O_2_ for 10 min. Subsequently, sections were treated with citrate buffer for 3 min (5 times), followed by blocking with 5% goat serum for 30 min (room temperature). Primary incubation was performed with rat anti-mouse CD31 (PECAM-1 - CloneSZ31, Dianova), and with rabbit anti-aSMA (alpha-Smooth Muscle Actin - EMELCA) for primary incubation and with Alexa Fluor 488 goat anti-rabbit IgG (Dianova) and Alexa Fluor 594 goat anti-rat IgG (Dianova) for secondary incubation, respectively. Primary incubation was performed overnight at 4 °C while secondary incubation was performed for 1 h at room temperature. p62 staining was performed using rabbit anti p62 (abcam ab91526) for primary incubation (4 °C, overnight), and with anti rabbit 488 (Jackson ImmunoResearch) for secondary incubation (1 h at RT). To visualize the nuclei, tissue sections were counterstained with DAPI.

### Statistics

All data were initially tested for normality using the Kolmogorov-Smirnov test. Normally distributed data were compared using the student’s ttest, not normally distributed data were analyzed using the Mann-Whitney test. Comparisons were always made between two groups. All results are given as mean +/−SEM. A *p*-value of below 0.05 was considered significant.

## Results

**Fig. 1:**
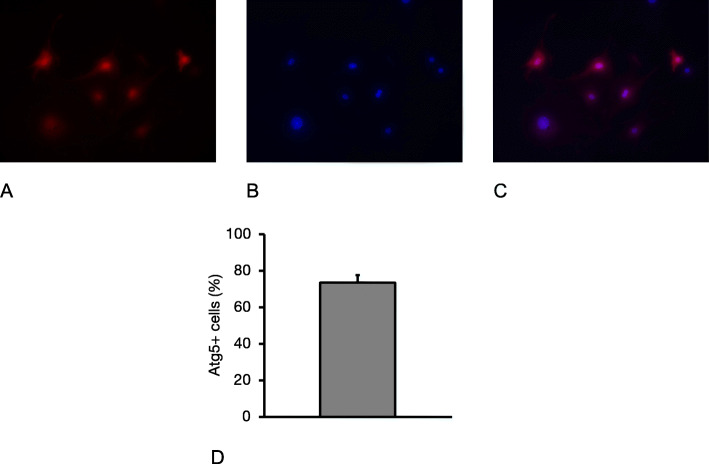
Evaluation of the Atg5 transfection rate. View field analysis revealed that 73.1 ± 4.6% of the cells showed red fluorescence, indicative for successful transfection. **a**: red signal indicates Atg5 expression; **b**: DAPI staining (nuclei); **c**: merge (magnifications in **a** – **c**: × 40; Data in **d** as mean +/− SEM)

**Fig. 2:**
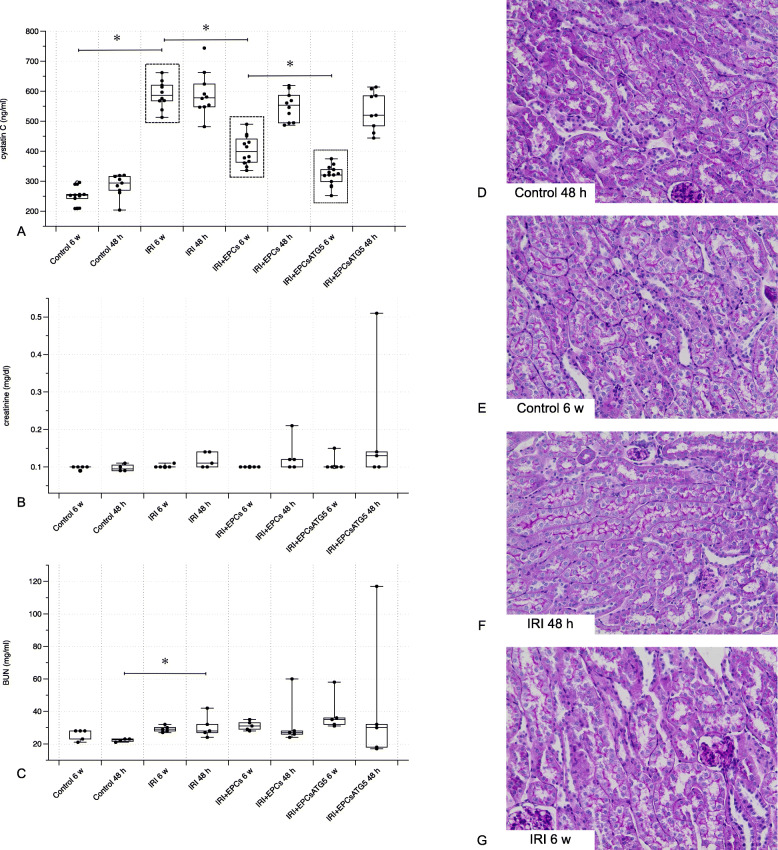
Evaluation of kidney excretory function and morphology. The bar diagrams **a-c** depict the results from three analyzes: serum cystatin C, serum creatinine, and blood urea nitrogen (BUN). Bilateral renal ischemia (IRI – Ischemia Reperfusion Injury) induced significant excretory kidney dysfunction as indicated by elevated serum cystatin C. While cell therapy failed to improve kidney function in all ‘48 h’ groups, kidney function improved at week 6, particularly after the administration of transfected cells. Regarding the analyzes of creatinine and BUN, only one difference reached the level of statistical significance: ‘Control 48 h’ vs. ‘IRI 48 h’. Images **d-g** show representative areas of tissue sections from the cortex. The pathological findings were mild overall and therefore did not allow a systematic comparison of structural differences (magnifications in **d**-**g** × 40; Data as mean +/− SEM, *: *p* < 0.05)

**Fig. 3:**
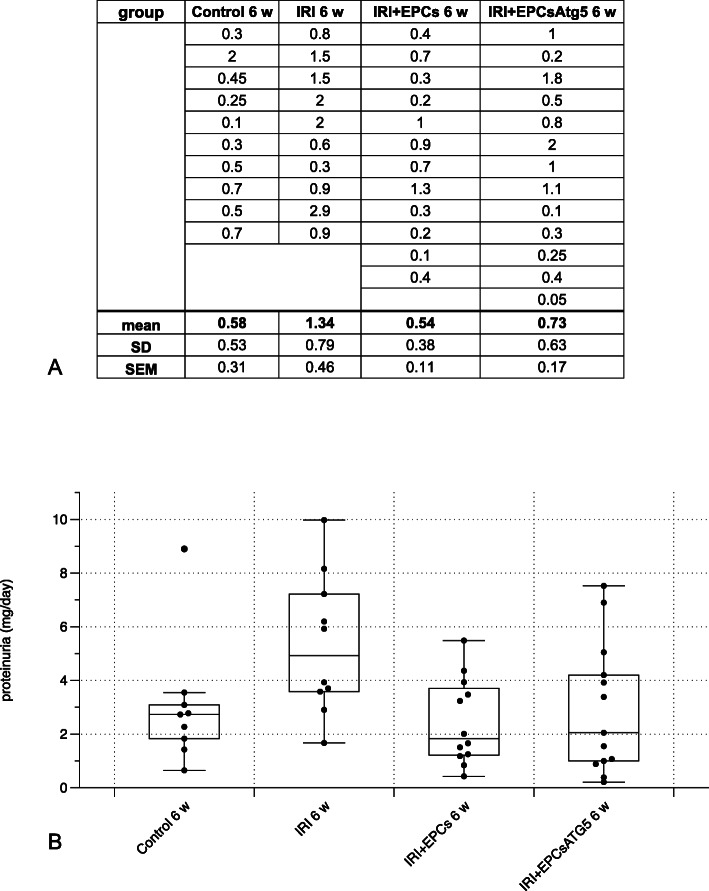
Proteinuria in all ‘6 weeks’ groups. Protein excretion was significantly elevated after ischemia and decreased as a results of cell therapy. Anti-proteinuric effects were observed in the presence of both, native and transfected cells. **a** shows the respective urine volumes of all animals analyzed, **b** illustrates the respective means +/−SEM of all four groups (Data as mean +/− SEM, *: *p*-value between ‘Control 6 w’ and ‘IRI 6 w’ < 0.05; #: *p*-value between ‘IRI 6 w’ and either ‘IRI + EPCs 6w’ or ‘IRI + EPCsAtg5 6w’ < 0.05)

**Fig. 4:**
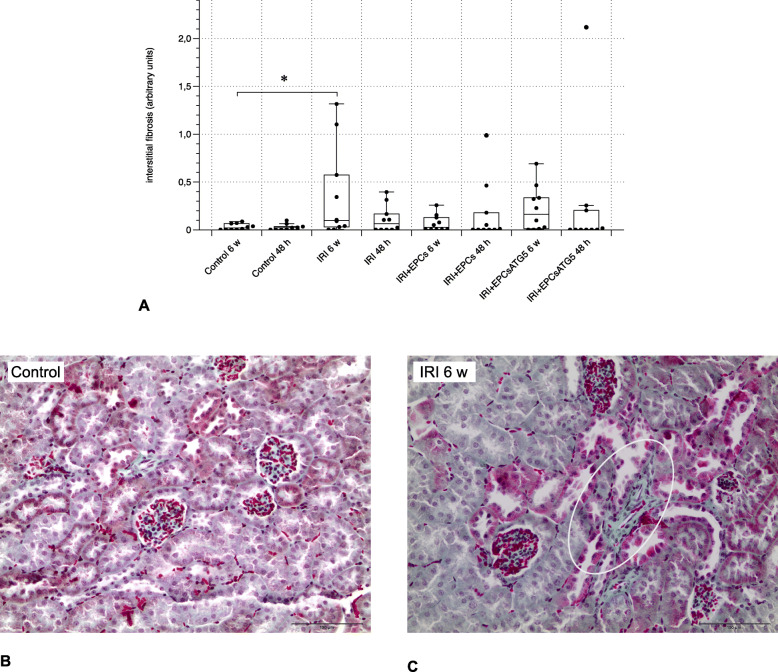
Interstitial fibrosis, evaluated after masson trichrome staining. Significant matrix deposition occurred exclusively in one group: ‘IRI 6 w’. All other groups did not differ between each other or in comparison to the control groups. The white oval in **c** surrounds an area of higher matrix density (magnifications in **a – c**: × 40; Data in **d** as mean +/− SEM)

**Fig. 5:**
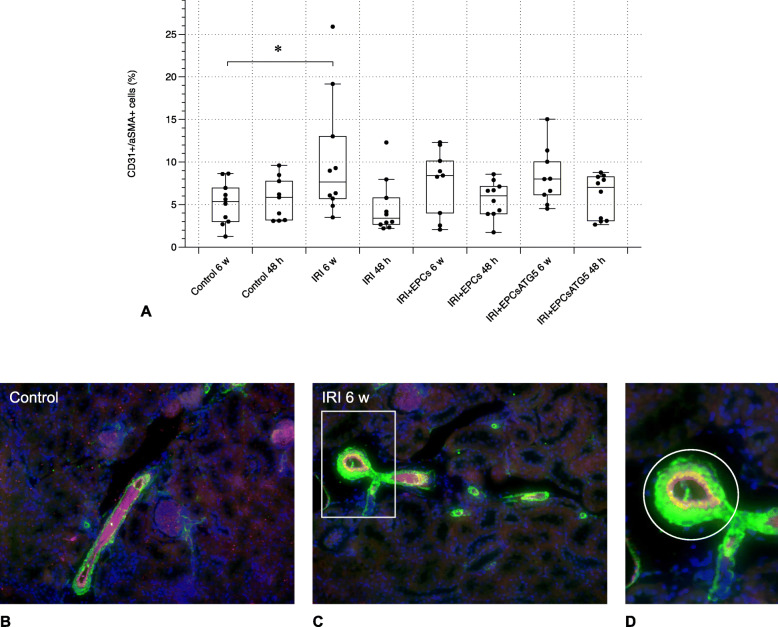
Endothelial-to-mesenchymal transition. Comparably to the fibrosis patterns, only one group (‘IRI 6 w’) showed different aSMA abundances in CD31+ cells. EPC treatment, either performed with native or transfected cells, did not modulate endothelial aSMA in a significant manner. Image **d** magnifies the surrounded (white rectangular) area in **c**. The white circle in **d** surrounds the borderzone between media (musculature - green) and intima, predominantely represented by the endothelial layer (red). The yellow indicates aSMA expression by CD31+ cells (magnifications in **b** and **c**: × 40, in **d** ∼ × 160; Data in **a** as mean +/− SEM; *: *p*-value between ‘Control 6 w’ and ‘IRI 6 w’ < 0.05)

**Fig. 6:**
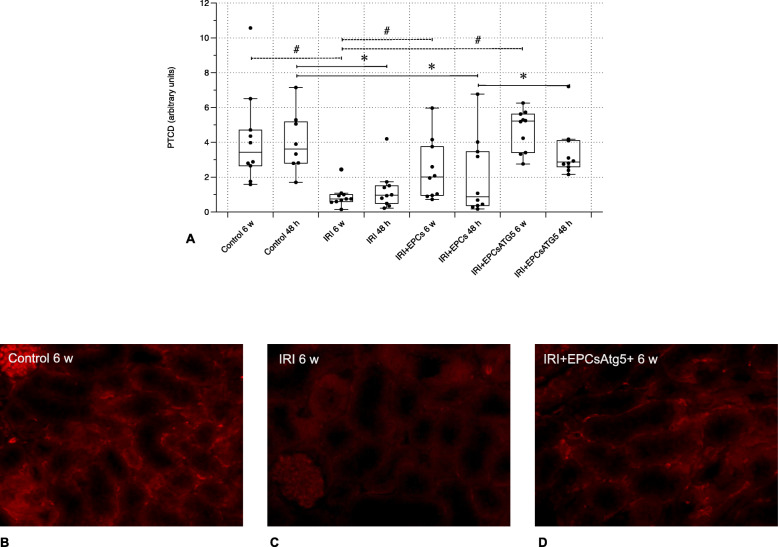
Peritubular capillary density (PTCD), evaluated after CD31 staining. IRI resulted in decreased PTCD at 48 h and 6 weeks, respectively. Native (non-transfected) EPCs increased the PTCD at 6 weeks but not earlier. Transfected cells however prevented the loss of capillaries completely (48 h and 6 weeks) (magnifications in **b-d** × 40; Data in **a** as mean +/− SEM; *: *p*-values between different groups of the ‘48 h cohort’ < 0.05 – the bracketed lines indicate comparisons between two groups; #: *p*-values between different groups of the ‘6 weeks cohort’ < 0.05 – the bracketed lines indicate respective comparisons)

**Fig. 7:**
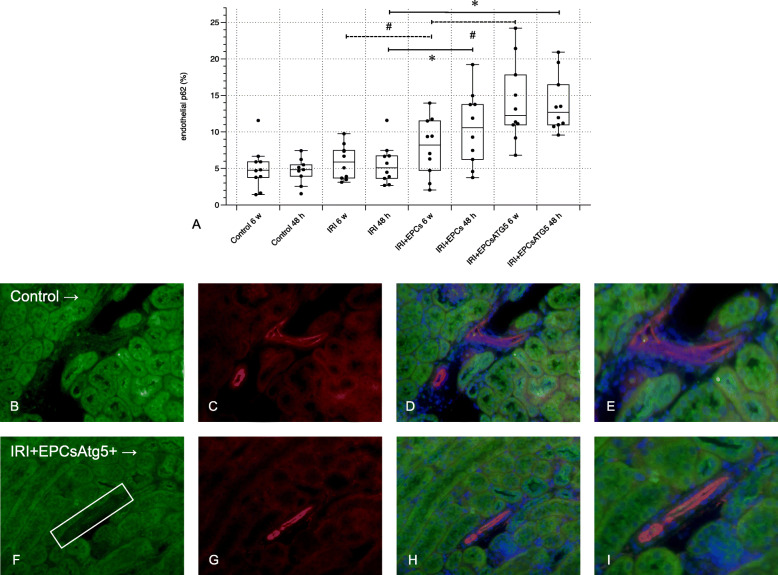
Analysis of the p62 expression in CD31+ (endothelial) cells. As in previous reports by our group, the autophagocytic flux was evaluated by p62 staining. IRI alone did not elevate endothelial p62. If native of transfected cells were applied however, endothelial p62 increased at both, 48 h and 6 weeks. Images **b**-**e** show kidney sections from one Control group (48 h), **f**-**i** display sections from an animal that received transfected EPCs (48 h). **b** + **f**: staining of p62 exclusively; **c** + **g**: staining of CD31 exclusively; **d** + **h**: merge; **e** + **i**: magnifications of **d** and **h**. (magnifications in **b-d** and in **f-h** × 40; in **e** and **i** ∼ × 60; green: p62; red: CD31; blue: nuclei; Data in A as mean +/− SEM; *: *p*-values between certain groups of the ‘48 h cohort’ < 0.05 – the bracketed lines indicate comparisons between two groups; #: *p*-values between different groups of the ‘6 weeks cohort’ < 0.05 – the bracketed lines indicate respective comparisons)

In the following paragraphs we will avoid to supply the respective numerical values of the analyzes if possible. All findings are depicted in Figs. 1, 2, 3, 4, 5, 6 and 7. Before the results will be presented, it needs to be stated that significant adverse events were not observed in any of the experimental groups. The mortality rate was 0%. Therefore, no animals required euthanization before all analytes were collected.

### Atg5 is constitutively expressed by transfected murine EPCs

After EPC transduction according to the protocol, 73.1 ± 4.6% of the cells showed red fluorescence, indicative for successful Atg5 expression (Fig. 1).

### EPCs^Atg5+^ improve post-ischemic serum cystatin C levels

Bilateral renal ischemia resulted in a significant increase in plasma cystatin C at 48 h. Cystatin C levels remained elevated until week 6. In all ‘48 h groups’, cell therapy did not substantially modulate concentrations of the metabolite. At 6 weeks however, EPC administration improved persisting kidney dysfunction. Native cells reduced cystatin C, this effect was even more pronounced if transfected cells (EPCs^Atg5+^) were applied. Together, the findings indicated a reno-protective role of EPCs that express Atg5 in a constitutive manner (Fig. 2). It needs to be mentioned that morphological analysis revealed only mild tubular damage (Fig. 2).

### EPCs and EPCs^Atg5+^ reduce proteinuria in the long-term

At week 6, post-ischemic animals displayed significant proteinuria as compared to untreated controls. Systemic injection of both, native and transfected cells diminished protein excretion with no difference between the two groups (Fig. 3). The figure also contains informations about the respective urine volumes that were collected and employed for analysis.

### Renal fibrosis and endothelial-to-Mesenchymal transition (EndoMT) are not significantly reduced in EPCs- or EPCs^Atg5+^-treated post-ischemic mice

Both, significant matrix deposition and endothelial expression of the mesenchymal marker alpha-Smooth Muscle Actin (aSMA) occurred exclusively in one out of 6 post-ischemic groups: ‘IRI 6 w’. Cell therapy was not reflected by any modulatory effect on neither fibrosis nor EndoMT at all (Figs. 4 and 5).

### EPCs^Atg5+^ completely prevent the kidney from peritubular capillary loss

Renal ischemia significantly reduced the peritubular capillary density (PTCD) at 48 h and 6 weeks. The administration of native (non-transfected) EPCs increased the PTCD at 6 weeks but not earlier. Transfected cells in contrast completely halted the loss of capillaries at both timepoints (Fig. 6).

### EPCs and EPCs^Atg5+^ augment the autophagocytic flux in renal endothelial cells

As in previous studies we evaluated the autophagocytic flux in endothelial cells by p62 staining [18]. Ischemia alone did not elevate endothelial p62. If native of transfected cells were applied, endothelial p62 increased at 48 h and 6 weeks (Fig. 7).

## Discussion

The current study substantially proves reno-protective effects of therapeutically administered EPCs that express Atg5 in a constitutive manner. Firstly, transfected cells improved persisting kidney dysfunction several weeks after ischemia and significantly reduced urinary protein excretion. Secondly, structural endpoints were modulated as well. Severe tubular damage did not occur. However, while interstitial matrix deposition and mesenchymal transdifferentiation of endothelial cells, both occuring at week 6, were not halted, transfected cells completely prevented the loss of peritubular capillaries. The latter has been identified as hallmark of acute ischemia and as risk factor for chronic kidney disease [20]. Administration of transfected cells also increased endothelial p62 abundances, indicative for an increased autophagocytic flux [13, 18].

In the past, we attempted to activate the autophagocytic cascade in EPCs by pharmacological measures exclusively. In total, four substances were evaluated, none of these reliably improved protective effects of the cells in AKI [13, 14]. We assumed that the lack of specificity of such drug-based approaches most likely accounted for the findings reported in previous studies. Cell-based therapies of ischemic and inflammatory diseases have increasingly been investigated in recent years. Most studies have been performed in animals, however, particularly mesenchymal stem cells (MSCs) are currently being evaluated in human diseases including AKI [21, 22]. In parallel, the mechanisms of therapeutic cell actions within the (post)ischemic or inflammatory microenvironment have gained the interest of researchers worldwide. Regarding MSCs and EPCs, two mechanisms have been identified, the release of certain types of vesicles and paracrinic actions, mediated by humoral factors or the so-called secretome. For more informations regarding EPCs, we would like to refer to the literature [5, 6]. Very recent and yet unpublished data from our laboratory indicated that intact EPCs must home to the postischemic kidney in order to promote AKI protection. In fact, systemically injected microvesicles protected the kidney as well but these effects occurred excusively under very defined conditions. Previous studies revealed that AKI protection mediated by intact EPCs can be stimulated with several substances such as Angiopoietin-1 and -2, and the hormone melatonin [4, 8, 9]. In our latest and yet unpublished series of experiments, such stimulatory effects were missing if microvesicles were injected alone. Since pharmacological strategies for AP activation failed so far and we generally strive to augment AKI-protective EPC effects in a persistent manner, genetic modification (herein: Atg5 transfection) became a promising option. To date, only few studies addressed the stimulation of autophagy in EPCs for therapeutic purposes. Zhou and colleagues [23] employed hypoxic preconditioning and found enhanced survival of the cells in a rat model of limb ischemia. Hu et al. [16] described reduced EPC migration and tube formation upon Atg5 inactivation, the authors applied a highly selective strategy (Atg5 gene silencing). Comparable approaches with other cell types such as mesenchymal stem cells (MSCs) have not been reported so far, although autophagy activation in MSCs has been shown to augment cell competence under different experimental conditions [24–26]. The mechanisms by which constitutive Atg5 activation enables the cells to protect the kidney are speculative in nature. Since AP is regarded as self-defensive strategy under certain conditions [11], one may hypothesize that Atg5 transduction increases the lifespan of injected EPCs or their stress-resistance within the post-ischemic microenvironment. However, our approach did not ensure a reliable labelling of EPCs, making any distinct conclusion about longer intra-renal engraftment impossible.

Although selective AP activation appears as promising option for enhancing EPC competence in cell-based AKI therapy, several problems remain. The two most important problems are related to the source of EPCs or other cell-types and the timing of cell administration. These aspects have recently been discussed [22].

In summary, we showed for the first time that constitutive Atg5 expression in therapeutically administered EPCs can potentially augment AKI protective effects of the cells. The potential consequences for the clinical management of the syndrome need to be defined.

## Conclusion

Constitutive Atg5 expression in therapeutically administered EPCs substantially improve AKI protective effects of the cells. Cell therapy improves both, excretory kidney function and structural endpoints. The consequences for the clinical management need to be determined.

## Data Availability

Data are available from the authors upon reasonable request (daniel.patschan@mhb-fontane.de).

## Abbreviations

AKI: Acute Kidney Injury
AP: Autophagy
aSMA: alpha-Smooth Muscle Actin
Atg5: Autophagy Related Protein5
EndoMT: Endothelial-to-Mesenchymal Transition
EPCs: Endothelial Progenitor Cells
PACs: Proangiogenic Cells
PTCD: Peritubular Capillary Density
SAHA: SuberoylAnilide Hydroxamic Acid
